# Supplementary material for: CUEDC2 modulates cardiomyocyte oxidative capacity by regulating GPX1 stability
Source: EMBO Mol Med. 2016 Jun 10;8(7):813–29. doi: 10.15252/emmm.201506010 (PMC4931293; doi:10.15252/emmm.201506010)
Supplement: Supplementary file 1 — Appendix [file EMMM-8-813-s001.pdf]

APPENDIX - CUEDC2 modulates cardiomyocytes oxidative capacity by regulating GPX1 stability

Zhao Jian, Bing Liang, Xin Pan, Guang Xu, Sai-Sai Guo, Ting Li, Tao Zhou, Ying-Bin Xiao, and Ai-Ling Li

Table of Contents

Appendix Figure S1 - The protein level of CUEDC2 was not changed after TAC…………2
Appendix Figure S2 - H/R treatment promoted CUEDC2 degradation……………………...3
Appendix Table S1 -Human samples associated clinical information……………………..…4
Appendix Figure S3 - CUEDC2 decreased under acute ischemic stimulation…………..…..5
Appendix Figure S4 - *CUEDC2* deletion has no apparent effect on heart structure, dimension and function………………………………………………………………………...….6
Appendix Table S2 - Echocardiography of dimensions and function in WT and *Cuedc2*$^{-/-}$ mice at physiological condition……………………………………………………….....7
Appendix Figure S5 - *Cuedc2* ablation alleviated oxidative stress and cell death in cardiomyocytes…………………………………………………………………………...8
Appendix Table S3 - Primers for qPCR…………………………………………………....9
Appendix Figure S6 - Loss of CUEDC2 had no obvious effect on ERK1/2 activation induced by I/R……………………………………………………………………………...10
Appendix Figure S7 - CUEDC2 knockout impaired the binding activity of NF-κB to its target following I/R……………………………………………………………………...11
Appendix Figure S8 - CUEDC2 destabilizes GPX1 by facilitating its ubiquitin/proteasome-dependent degradation……………………………………….....12
Appendix Figure S9 - CUEDC2 interacted with GPX1 and promoted GPX1 degradation..13
Appendix Figure S10 - The original gel result of the IPed complex used for mass spectrometry………………………………………………………………………………14
Appendix Table S4 - List of GPX1 binding proteins identified by mass spectrometry….....15
Appendix Figure S11 - The role of CUEDC2 on TRIM33 regulation and interaction of GPX1………………………………………………………………………………………16
Appendix Figure S12 - Immunoblotting for GFP protein level……………………………17
Appendix Figure S13 - Body weights (BW) of young (8 weeks) and old mice (20 months) mice……………………………………………………………………………………..18
Appendix Figure S14 - A scheme of the role of CUEDC2 in regulating GPX1 stability and I/R injury in heart……………………………………………………………………………19
Appendix Figure S15 - ER-alpha and PR protein levels had no differences in the hearts of WT and *Cuedc2*$^{-/-}$ mouse………………………………………………………….… 20

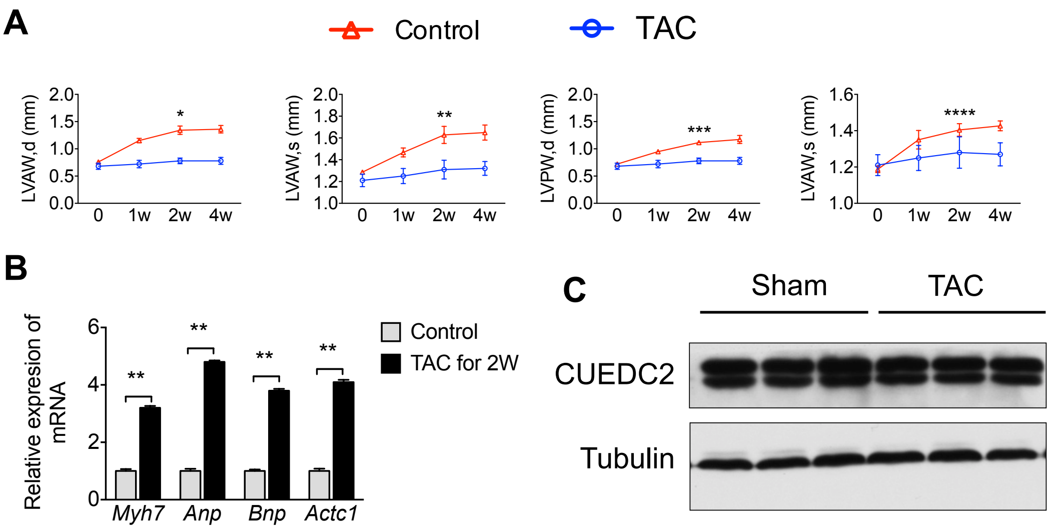

Appendix Figure S1. The protein level of CUEDC2 was not changed after TAC.

A The thickness of left ventricle was evaluated by transthoracic echocardiography at different time points post transthoracic aortic coarctation (TAC). LVAW, d, left ventricle anterior wall in diastole; LVAW,s, left ventricle anterior wall in systole; LVPW, d, left ventricle posterior wall in diastole; LVPW,s left ventricle posterior wall in systole; * $p = 0.0171$ in LVAW,d parameter; ** $p = 0.0213$ in LVAW,s parameter; *** $p = 0.0318$ in LVPW,d parameter; **** $p = 0.0285$ in LVPW,s parameter, n = 10 in each group) compared to the left ventricle thickness parameters tested just before TAC.

B Quantitative−PCR for the cardiac hypertrophy gene expressions. Gene expressions at 2 weeks post-TAC were compared with the control group. ** $p < 0.0001$, n = 10 in each group). *Myh7*, cardiac myosin heavy chain beta; *Anp*, atrial natriuretic peptide; *Bnp*, B-type natriuretic peptide; *Actc1*, cardiac muscle alpha actin.

C Protein was extracted from left ventricle from control mouse or from mouse 2 weeks post-TAC and subjected to western blotting.

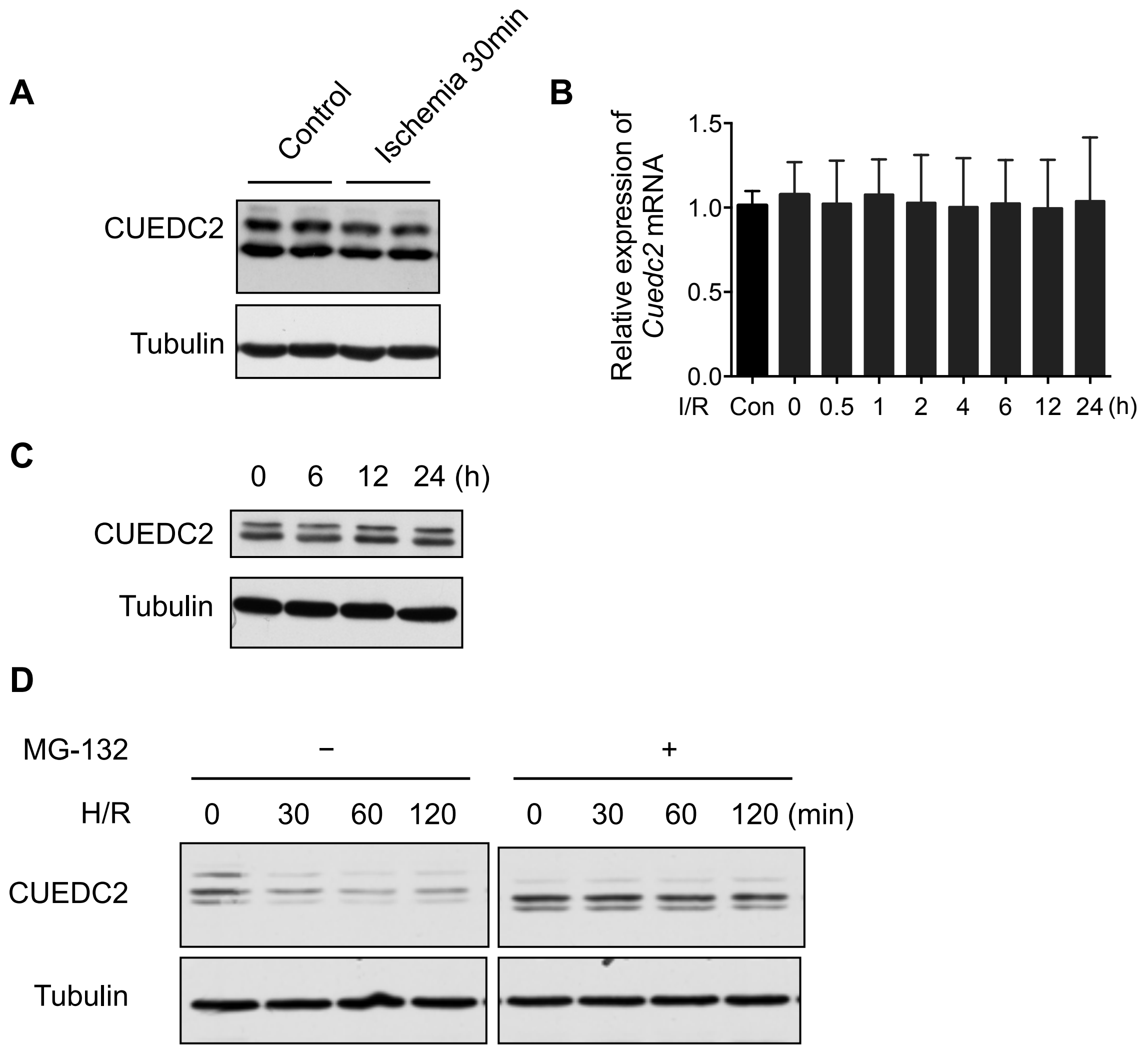

Appendix Figure S2. H/R treatment promoted CUEDC2 degradation.

A Protein was extracted from left ventricle in control group and area at risk 30 minutes post-ischemia group, and CUEDC2 was examined by immunoblot.

B RNA was extracted from area at risk post-I/R at different time points. And quantitative PCR was performed to test *CUEDC2* mRNA expression level. Con, control group without any treatment.

C Neonatal mouse cardiomyocytes were isolated and cultured in the same condition without the treatment of H/R. Total protein was harvested and protein level of CUEDC2 was examined at different time points.

D CUEDC2 was degraded by H/R treatment in a proteasome-dependent way. Neonatal mouse cardiomyocytes were firstly treated or not with MG-132 (10uM, 4hrs), and subjected to hypoxia (0.3% oxygen) with serum−free medium for 6h and then reoxygenated with normal concentration of oxygen and normal growth medium for another 2 hours. Protein was extracted for immunoblot analysis at the times indicated.

Appendix Table S1. Human samples associated clinical information.

Acute myocardial infarction

No.	Diagnosis	year	sex	localization
1	Acute infarction	81	female	anterior
2	Acute infarction	68	female	posterior
3	Acute infarction	71	male	septal
4	Acute infarction	48	female	posterior
5	Acute infarction	45	male	anterior
6	Acute infarction	51	male	septal
7	Acute infarction	44	female	anterior
8	Acute infarction	68	male	septal
9	Acute infarction	83	male	anterior

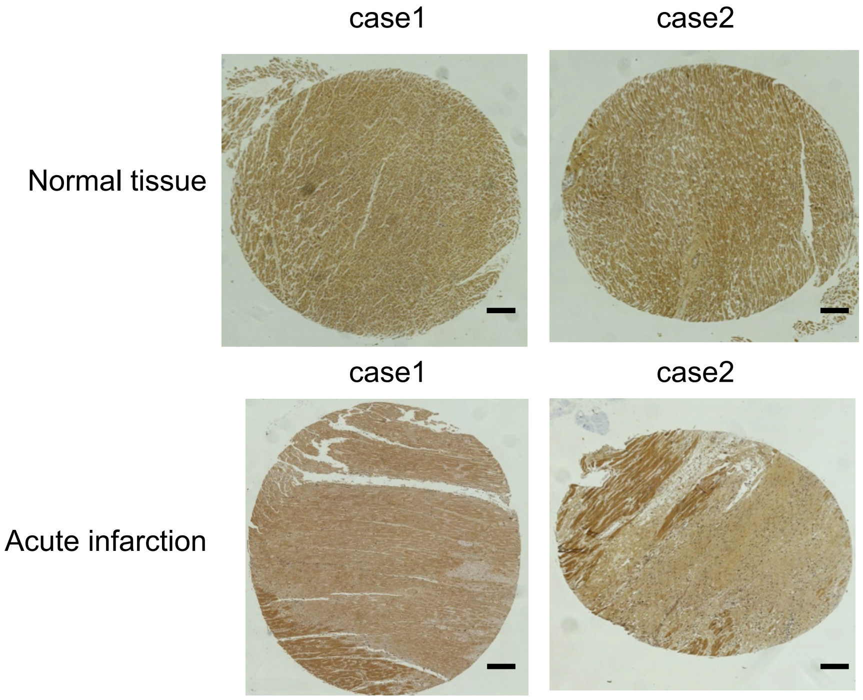

Appendix Figure S3. CUEDC2 decreased under acute ischemic stimulation.
Representative images from immunohistochemical staining of CUEDC2 in tissues from patients suffered with myocardial infarction. Heart samples obtained from donors who died from neurological diseases or motor-vehicle accidents were used as normal control.

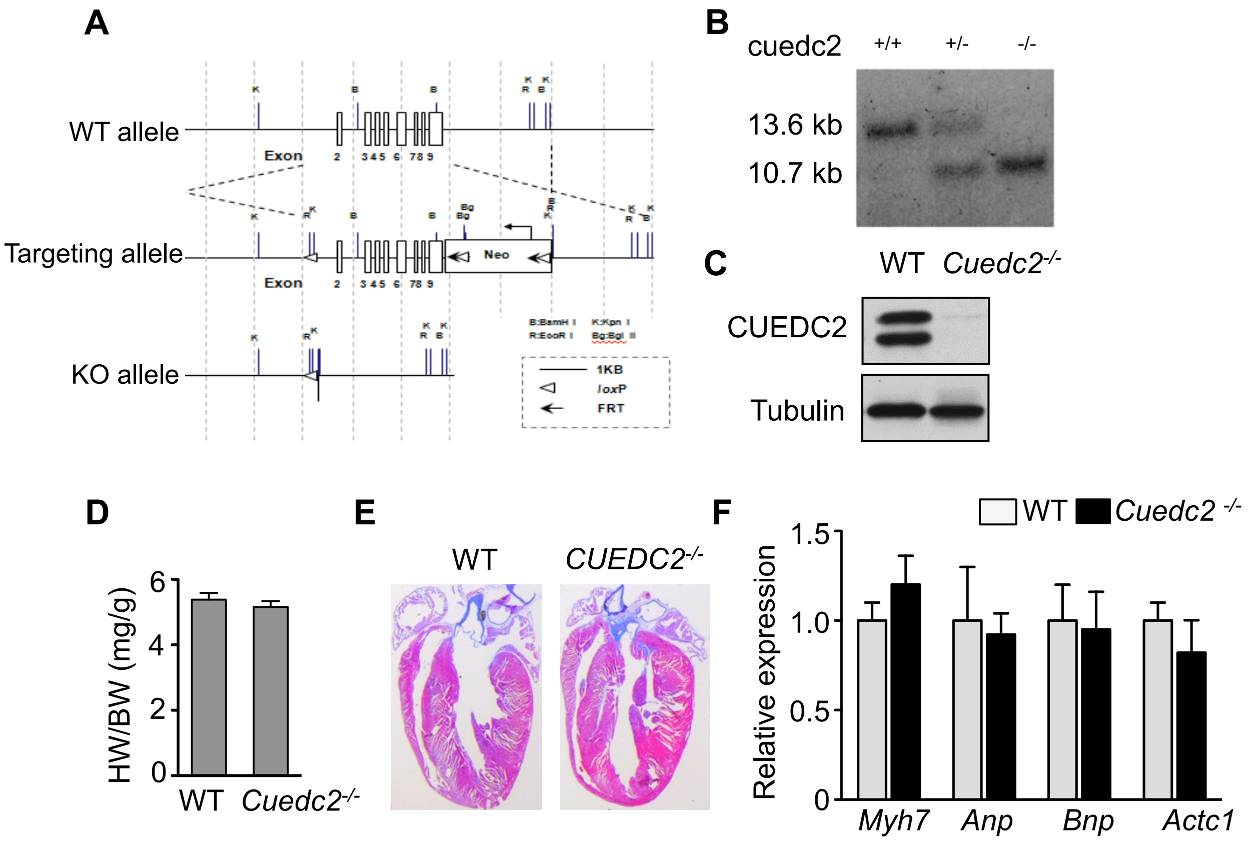

Appendix Figure S4. ***CUEDC2*** **deletion has no apparent effect on heart structure, dimension and function.**

A The strategy of *Cuedc2* knockout in mouse. *Cuedc2*$^{-/-}$ mice were generated by homologous recombination. The targeting vector containing a neomycin cassette was introduced in place of exons 2 to 9 of *Cuedc2*.

B Confirmation of *Cuedc2* deletion in mouse heart by northern blot.

C Confirmation of *Cuedc2* deletion in mouse heart by immunoblotting.

D Heart weight (HW) to body weight (BW) ratio from 8-week-old WT and *Cuedc2*$^{-/-}$ mice.

E Representative staining of histological sections in WT and *Cuedc2*$^{-/-}$ mice hearts by Masson staining. Scale bar, 50 μm.

F Quantitative-PCR expression analysis of cardiac stress response genes in *Cuedc2*$^{-/-}$ mice heart relative to WT. n = 3 per group; data are representative of 2 separate experiments. Data are representative of two separate experiments. Data are shown as mean ± SEM.

Appendix Table S2. Echocardiography of dimensions and function in WT and *Cuedc2*$^{-/-}$ mice at physiological condition.

	WT baseline	*Cuedc2*$^{-/-}$ baseline
BW (g)	20.3 ± 2.1	21.1 ± 1.9
HW/BW(mg/g)	5.7 ± 1.3	5.4 ± 1.6
LV mass index (mg)	114.3 ± 10.6	110.5 ± 11.2
HR (bpm)	424.15 ± 26.43	418.22 ± 22.26
LVAW,d (mm)	0.76 ± 0.08	0.72 ± 0.06
LVAW,s (mm)	1.29 ± 0.06	1.23 ± 0.07
LVPW,d (mm)	0.72 ± 0.06	0.72 ± 0.05
LVPW,s (mm)	1.19 ± 0.07	1.12 ± 0.06
LVST,d (mm)	0.83 ± 0.05	0.81 ± 0.07
LVST,s (mm)	1.32 ± 0.12	1.28 ± 0.15
LVID,d (mm)	4.12 ± 0.21	4.03 ± 0.18
LVID,s (mm)	2.65 ± 0.06	2.59 ± 0.04
FS (%)	35.35 ± 3.12	36.21 ± 2.85
EF (%)	64.87 ± 2.67	66.26 ± 2.92

Transthoracic echocardiography on anesthetized mice. Data are mean ± SEM. HR, heart rate; LVAW,d, end-diastolic LV anterior wall thickness; LVAW,s, end-systolic LV anterior wall thickness; LVPW,d, end-diastolic LV posterior wall thickness; LVAW,s, end-systolic LV posterior wall thickness; LVST,d, interventricular septal thickness in diastole; LVST,s, interventricular septal thickness in systole; LVID,d, diastolic left ventricular internal diameters; LVID,s, systolic left ventricular internal diameters; FS, fractional shortening; EF, ejection fraction. LV mass index was calculated as (external LV diameter in diastole^3 − LV end-diastolic dimension^3) × 1.055.

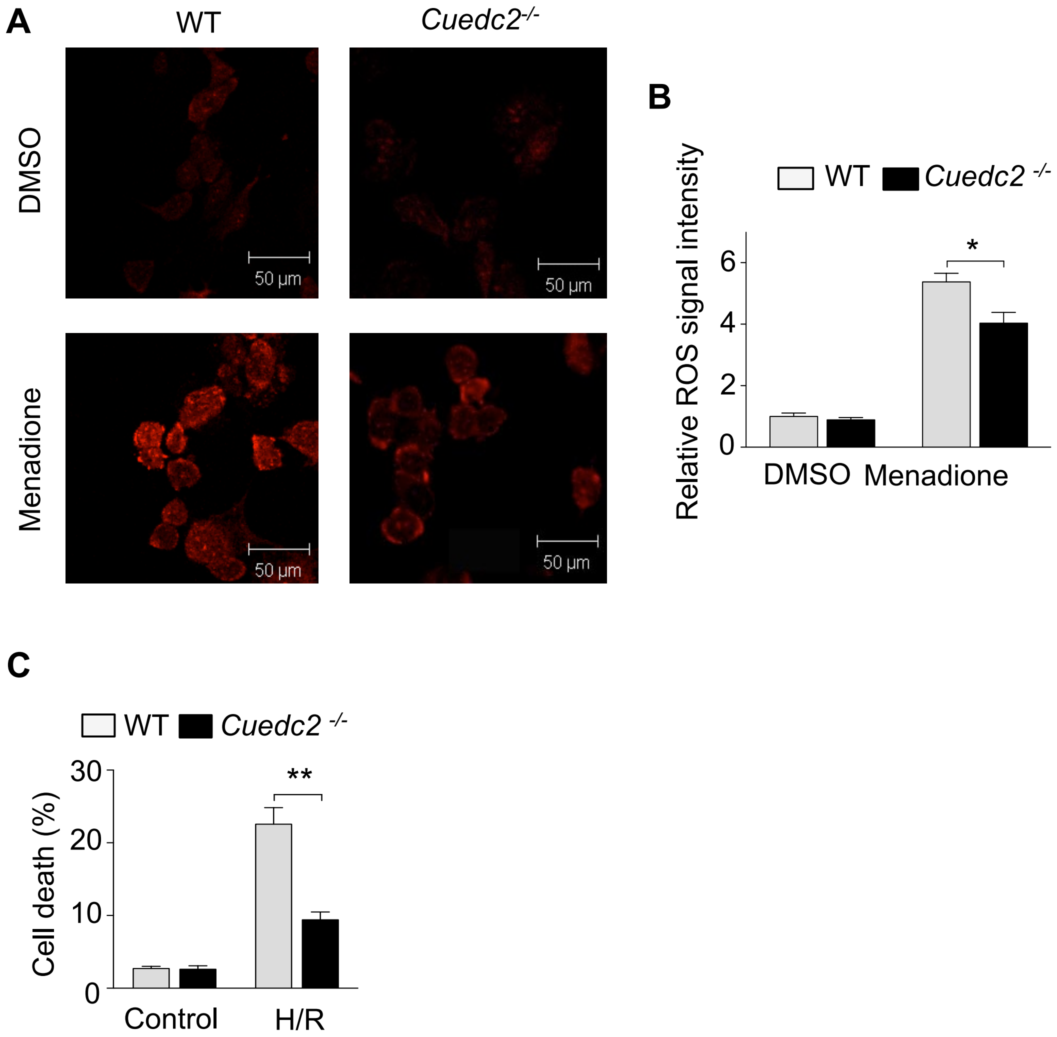

Appendix Figure S5. *Cuedc2* ablation alleviated oxidative stress and cell death in cardiomyocytes.
A, B Neonatal mouse cardiomyocytes were treated with or without 100 µM menadione for 1 hour at 37°C, then stained with CellROX® Deep Red Reagent and analyzed as in B. * $p = 0.0082$, n = 3 wells per group.
C Neonatal mouse cardiomyocytes were subjected to hypoxia for 6 hours with serum-free medium and then reoxygenation (21% oxygen) accompanying with adding back serum for 6 h, and then cardiomyocyte cell death was determined by flow cytometry. Quantitative analysis of PI-positive cells was shown (** $p = 0.0031$, n=5 wells per group and repeated three times).

Appendix Table S3. Primers for qPCR.

Gene	Forward primer	Reverse primer
TNFα	5'- GGGCAGTTAGGCATGGGAT-3'	5'-TGAGCCTTTTAGGCTTCCCAG-3'
IL-6	5'-CACTTCACAAGTCGGAGGCT-3'	5'-CTGCAAGTGCATCATCGTTGT-3'
IL-23	5'- CCAGCAGCTCTCTCGGAATC -3'	5'- CACTGGATACGGGGCACATT -3'
Myh7	5'- CTTGCTACCCTCAGGTGGCT -3'	5'- GAGCCTTGGATTCTCAAACG -3'
ANP	5'- GGGGGTAGGATTGACAGGAT -3'	5'- AGGGCTTAGGATCTTTTGCG -3'
Acta 1	5'- CTCACTTCCTACCCTCGGC -3'	5'- CAAAGCCAGCTTTCACCAG -3'
Bnp	5'- ACAAGATAGACCGGATCGGA -3'	5'- AAGAGACCCAGGCAGAGTCA -3'
GAPDH	5'- ATGTTCCAGTATGACTCCACTCACG -3'	5'- GAAGACACCAGTAGACTCCACGACA -3'

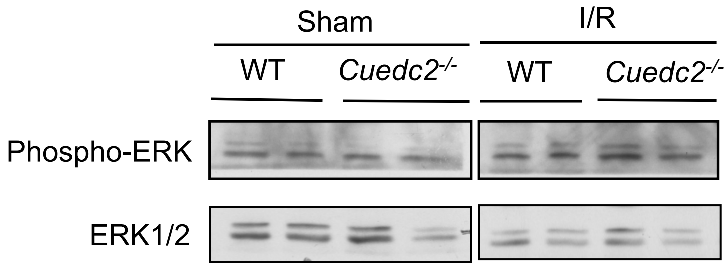

Appendix Figure S6. **Loss of CUEDC2 had no obvious effect on ERK1/2 activation induced by I/R.** Protein was extracted from the area at risk of left ventricle from WT of *Cuedc2*$^{-/-}$ mice with 30-minute ischemia followed by 30-minute reperfusion and subjected to immunoblot analysis. Representative results from 2 mice in each group were shown, and each experiment was repeated for three times.

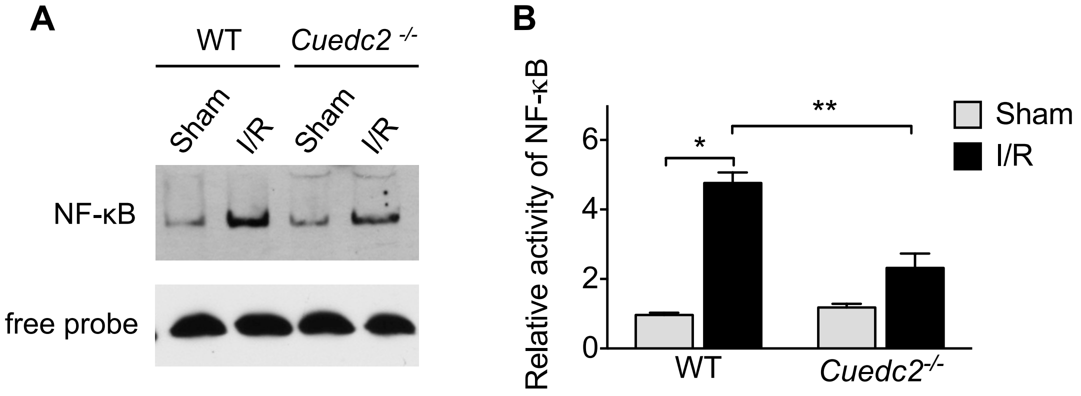

Appendix Figure S7. CUEDC2 knockout impaired the binding activity of NF-κB to its target following I/R.

A Nuclear extracts prepared from cardiac tissues of WT and *Cuedc2* $^{-/-}$ mouse under sham operation or I/R for 30 min were subjected to EMSA experiment. A representative result of three independent experiments.

B The statistical results of three independent experiments. * p = 0.0002, ** p = 0.0472, n = 3 in each group..

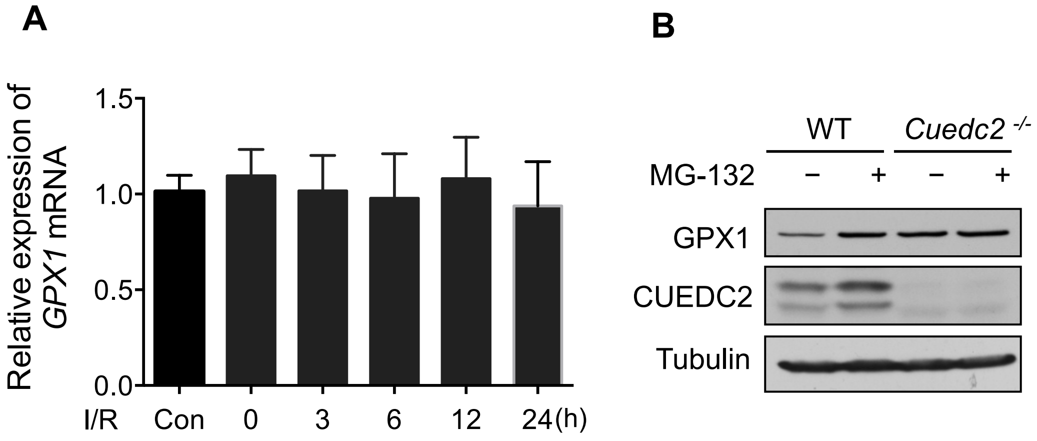

Appendix Figure S8. CUEDC2 destabilizes GPX1 by facilitating its ubiquitin/proteasome-dependent degradation.

A The mRNA expression level of GPX1 during I/R injury was analyzed by quantitative PCR.

B Primary neonatal mouse cardiomyocytes were treated with or without MG-132 (10 µM) for 6 hours, and then subjected to immunoblotting.

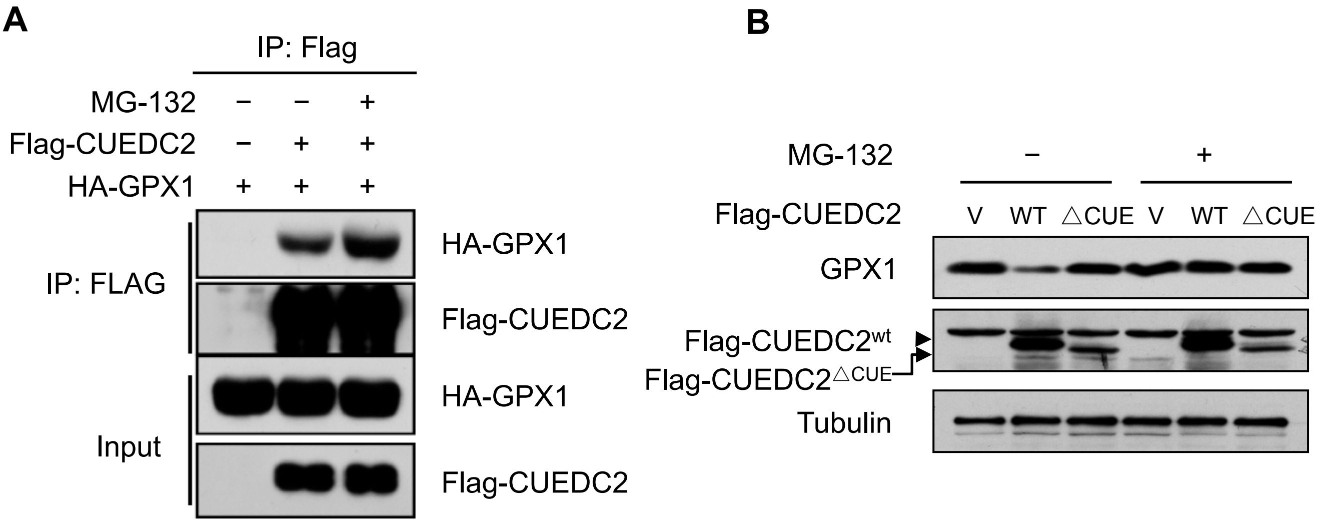

Appendix Figure S9. CUEDC2 interacted with GPX1 and promoted GPX1 degradation.

A The interaction was more robust in the presence of MG132. v: vector.

B HEK293T cells were transfected with the Flag-CUEDC2 or Flag-CUEDC2$^{\triangle CUE}$. At 24 hours after transfection, the cells were treated with or without MG-132 (10 μM). Cells were cultured for additional 6 hours and subjected to immunoblotting, and each experiment was repeated for three times.

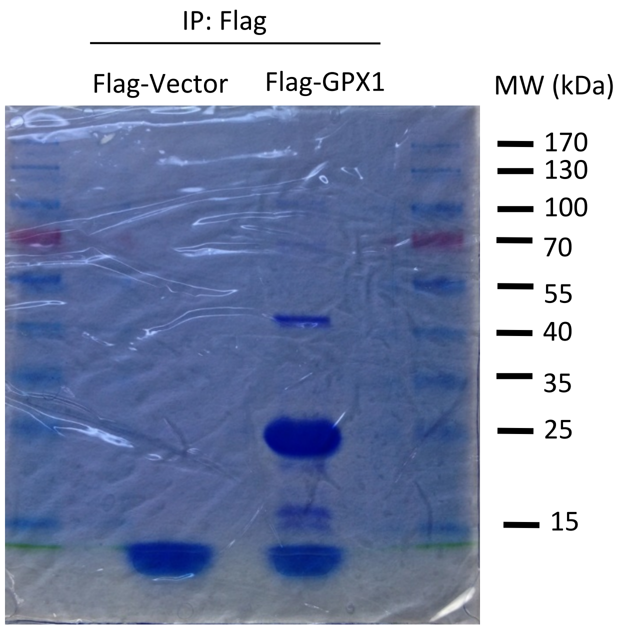

Appendix Figure S10. The original gel result of the IPed complex used for mass spectrometry.

Appendix Table S4. List of GPX1 binding proteins identified by mass spectrometry

Protein Name
polyubiquitin-B precursor [Homo sapiens]
tropomyosin alpha-3 chain isoform 2 [Homo sapiens]
thioredoxin isoform 1 [Homo sapiens]
tropomyosin alpha-1 chain isoform 6 [Homo sapiens]
EF-hand domain-containing protein D1 isoform 1 [Homo sapiens]
triosephosphate isomerase isoform 3 [Homo sapiens]
F-actin-capping protein subunit alpha-1 [Homo sapiens]
F-actin-capping protein subunit beta isoform 1 [Homo sapiens]
E3 ubiquitin-protein ligase TRIM33 isoform beta [Homo sapiens]
transmembrane protease serine 3 isoform 2 [Homo sapiens]
enhancer of rudimentary homolog [Homo sapiens]
CUE-domain containing protein 2 [Homo sapiens]
eukaryotic initiation factor 4A-I isoform 2 [Homo sapiens]
L-lactate dehydrogenase B chain [Homo sapiens]
dynein light chain 1, cytoplasmic [Homo sapiens]
splicing factor U2AF 26 kDa subunit isoform 1 [Homo sapiens]
malate dehydrogenase, mitochondrial precursor [Homo sapiens]
GTP-binding protein Di-Ras2 [Homo sapiens]
eukaryotic translation initiation factor 5A-2 [Homo sapiens]
drebrin-like protein isoform b [Homo sapiens]
synaptic vesicle membrane protein VAT-1 homolog [Homo sapiens]
occludin isoform b precursor [Homo sapiens]
ornithine aminotransferase, mitochondrial isoform 2 [Homo sapiens]
calcium release-activated calcium channel protein 1 [Homo sapiens]
fructose-bisphosphate aldolase A isoform 1 [Homo sapiens]

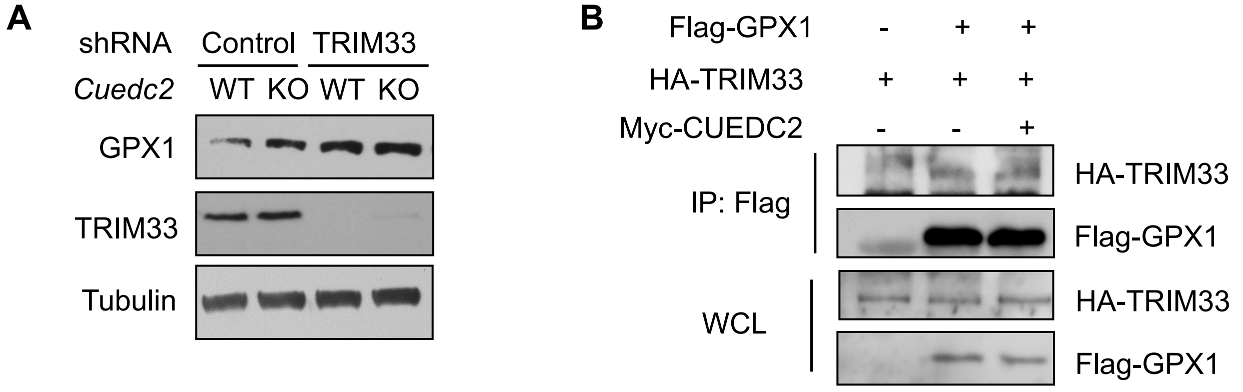

Appendix Figure S11. The role of CUEDC2 on TRIM33 regulation and interaction of GPX1.
A Wild-type or *Cuedc2*-/- cardiomyocytes were transfected with lentivirus carrying control or TRIM33 shRNA, 48 h post-transfection, the protein level of GPX1 was tested by immunoblotting. Each experiment was repeated for 3 times.
B HEK293T cells were transfected with indicated plasmids, and treated with MG-132 (10 µM) for 6 hours before harvest. Cell lysates were immunoprecipitated (IP) with anti-Flag (M2). The immunoprecipitates and the whole cell lysates (WCL) were analyzed by western blot.

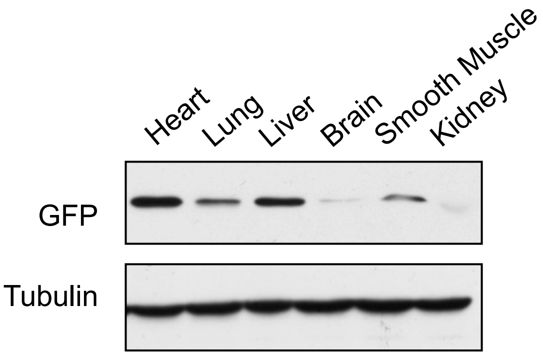

Appendix Figure S12. Immunoblotting for GFP protein level.
4 weeks after tail-vain injection of rAAV9-GFP, mice tissues were harvested and subjected to immunoblotting for GFP protein levels.

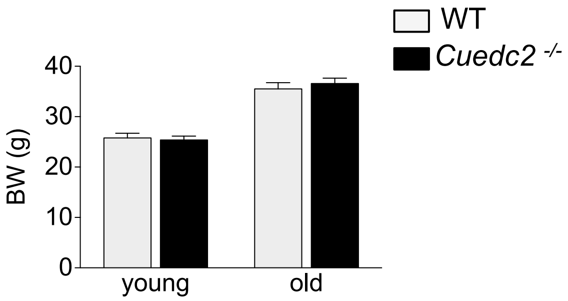

Appendix Figure S13. Body weights (BW) of young (8 weeks) and old mice (20 months) mice. n = 5 mice in WT group and n = 5 mice in *Cuedc2* $^{-/-}$ group.

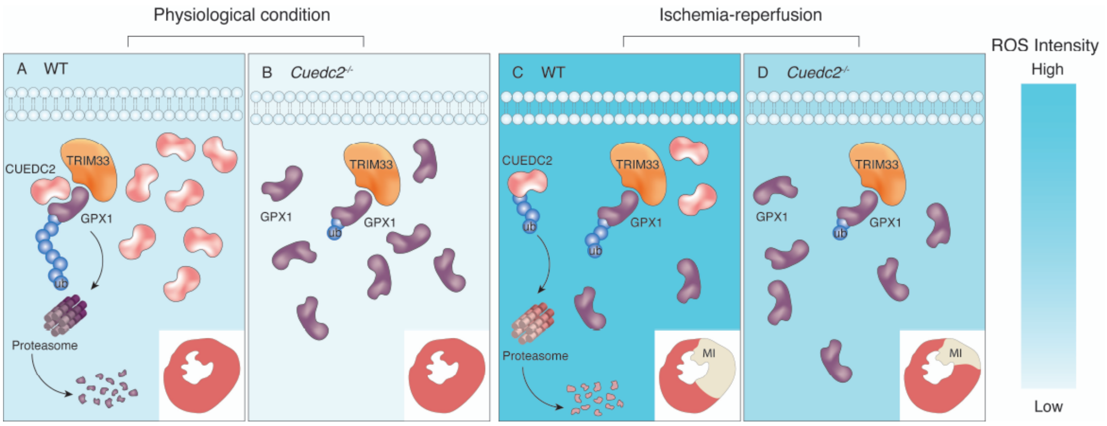

Appendix Figure S14. A scheme of the role of CUEDC2 in regulating GPX1 stability and I/R injury in heart.

A CUEDC2 was highly expressed in the heart under physiological condition.

B The protein level of GPX1 was increased as TRIM33-mediated ubiquitin-proteasome dependent degradation of GPX1 was decreased when *Cuedc2* was knocked out.

C I/R-induced injury, such as myocardial infarction (MI), is primarily triggered by the generation of massive amounts of ROS. Following I/R, CUEDC2 protein level gradually decreased which led to the increase in GPX1 protein level to scavenge ROS. Therefore, CUEDC2 degradation upon I/R is an intrinsic protective mechanism against I/R injury in heart.

D When CUEDC2 was ablated, GPX1 protein level dramatically increased in heart and I/R injury was alleviated.

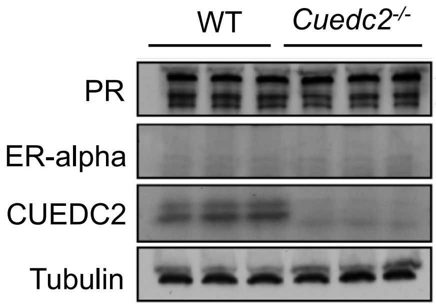

Appendix Figure S15. ER-alpha and PR protein levels had no differences in the hearts of WT and *Cuedc2*$^{-/-}$ mouse.

The protein level of ER-alpha and PR in WT and *Cuedc2*$^{-/-}$ heart. Total protein was extracted from the left ventricle of WT and *Cuedc2*$^{-/-}$ mice. Representative results from 3 mice are in each group were shown, and each experiment was repeated for three times.
