# Supplementary figures and images for: CUEDC2 modulates cardiomyocyte oxidative capacity by regulating GPX1 stability
Source: EMBO Mol Med. 2016 Jun 10;8(7):813–29. doi: 10.15252/emmm.201506010 (PMC4931293; doi:10.15252/emmm.201506010)

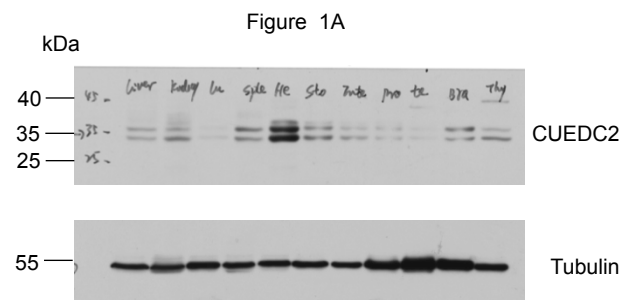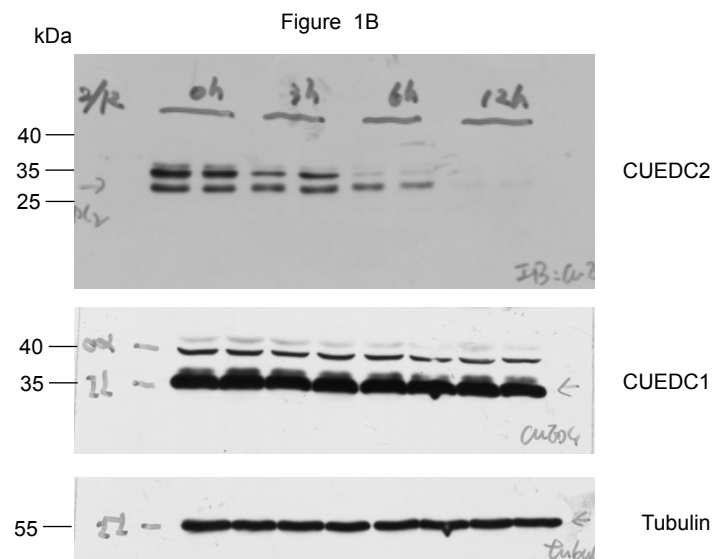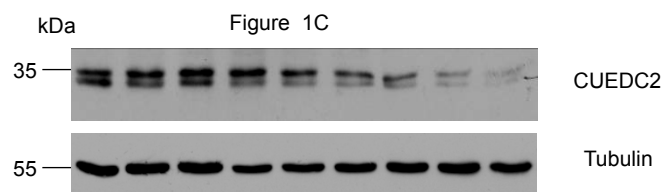

Supplement: Supplementary file 3 — Source Data for Figure 1 [file EMMM-8-813-s002.pdf]

Figure 3A

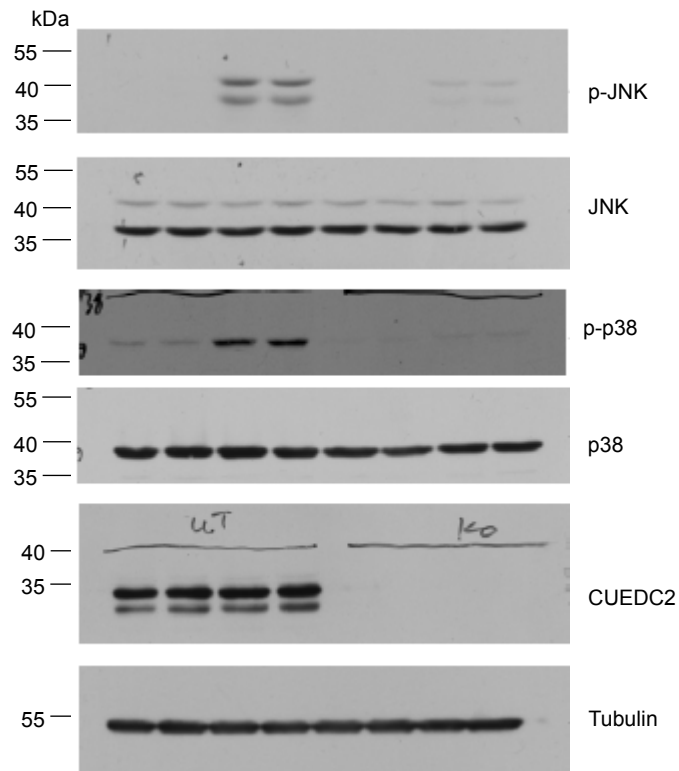

Figure 3B

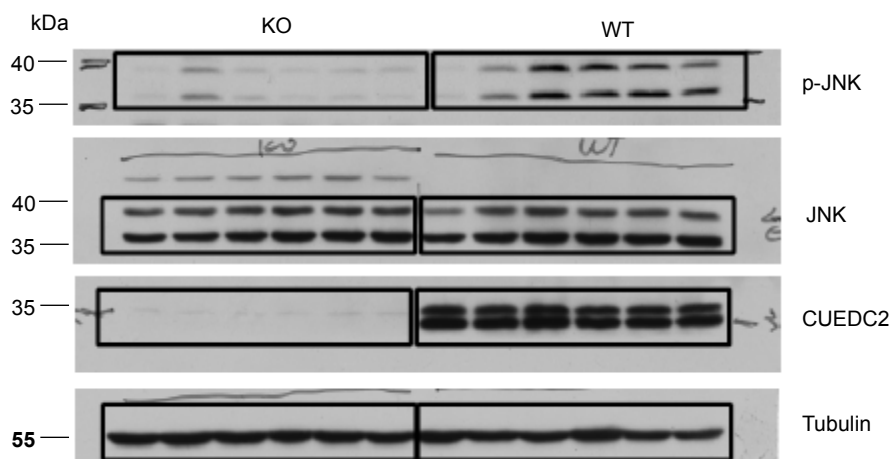

Supplement: Supplementary file 4 — Source Data for Figure 3 [file EMMM-8-813-s003.pdf]

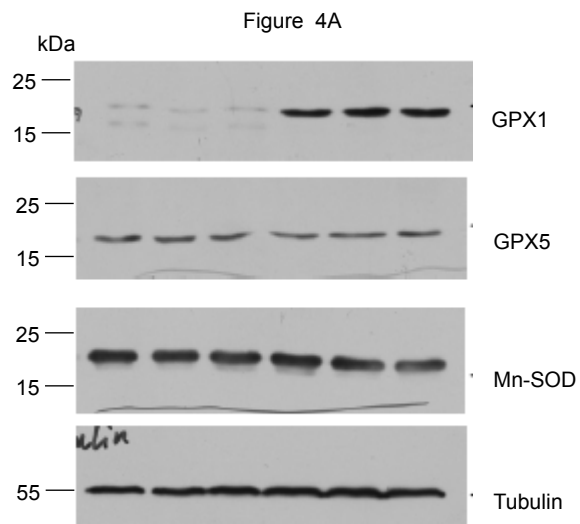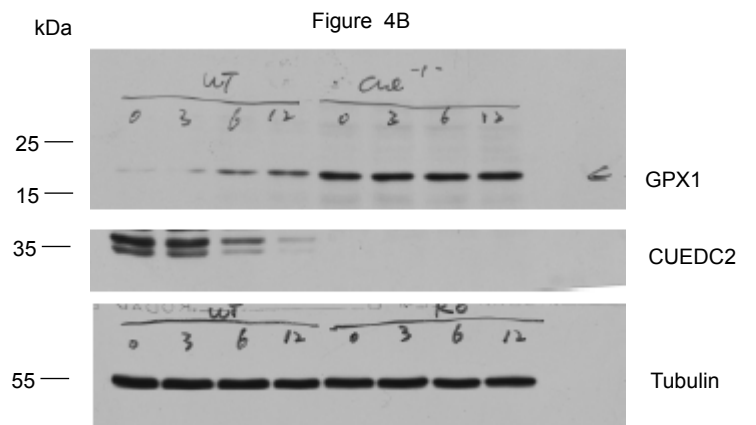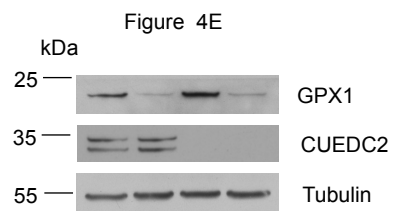

Supplement: Supplementary file 5 — Source Data for Figure 4 [file EMMM-8-813-s004.pdf]

Figure 5A

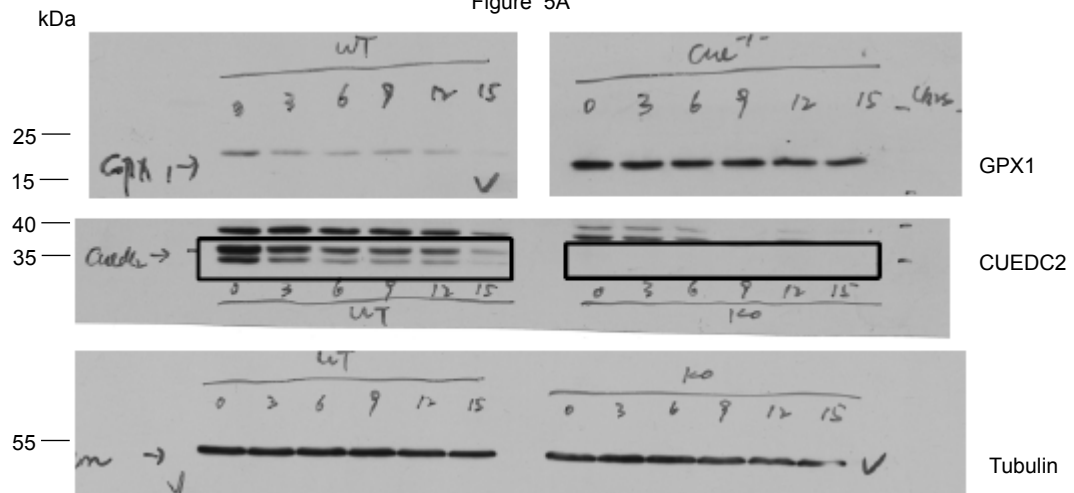

Figure 5B

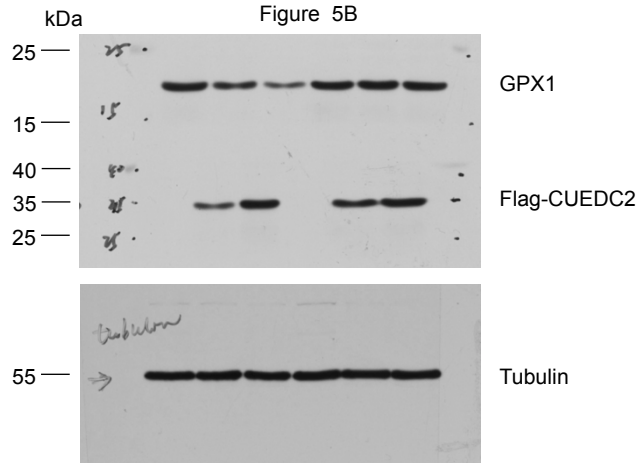

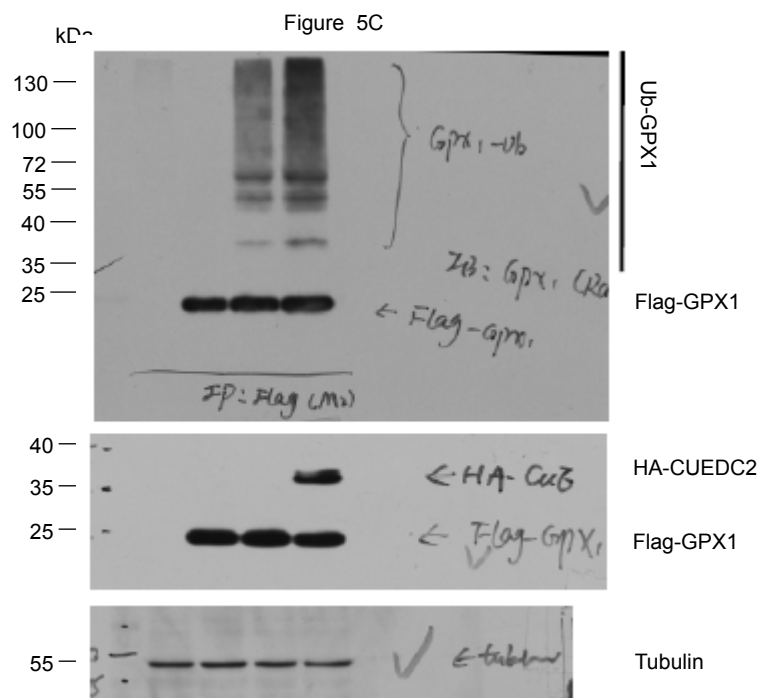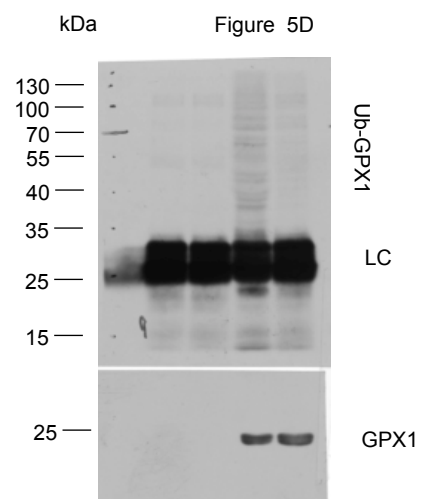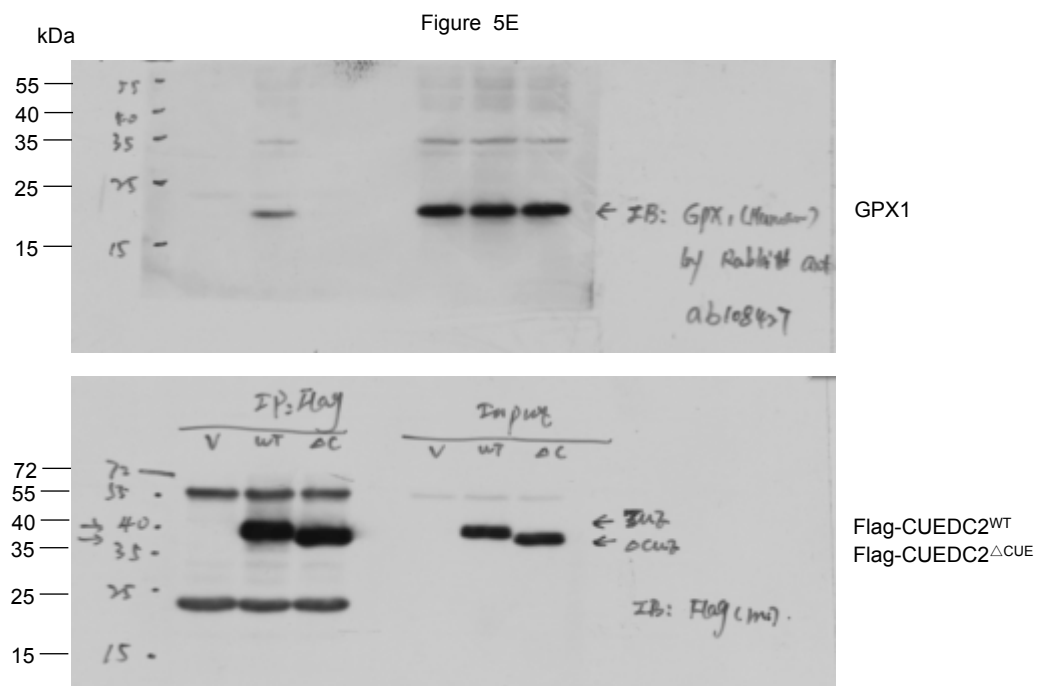

Supplement: Supplementary file 6 — Source Data for Figure 5 [file EMMM-8-813-s005.pdf]

Figure 6A

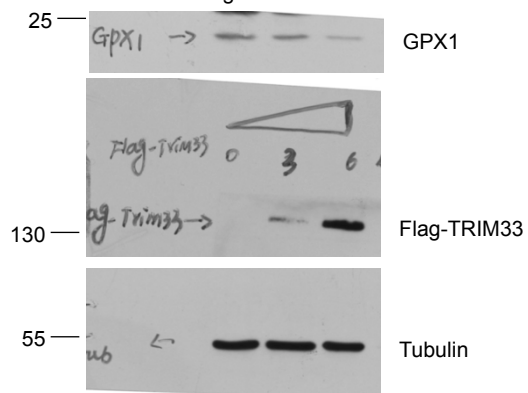

Figure 6B

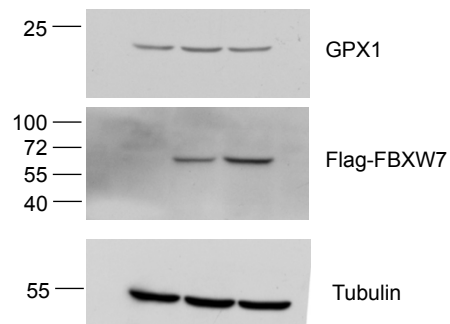

Figure 6C

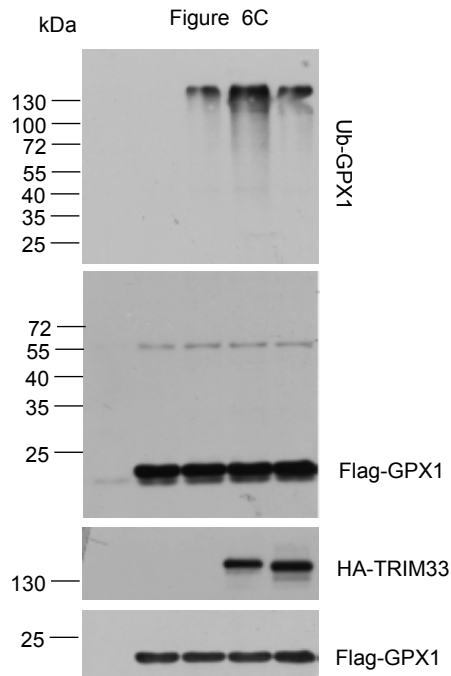

Figure 6D

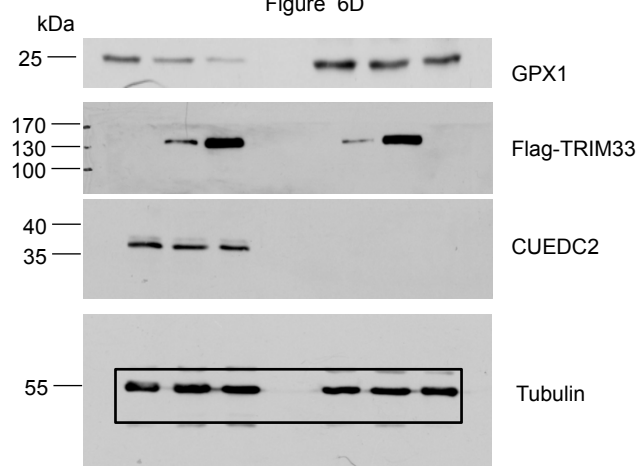

Supplement: Supplementary file 7 — Source Data for Figure 6 [file EMMM-8-813-s006.pdf]

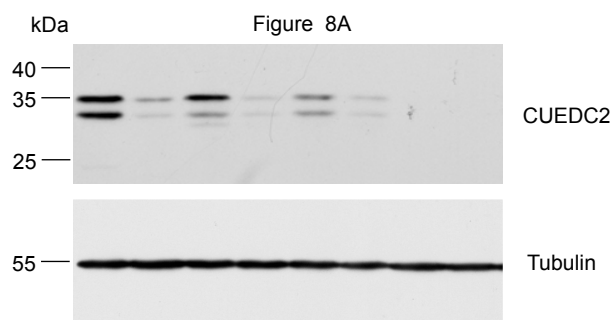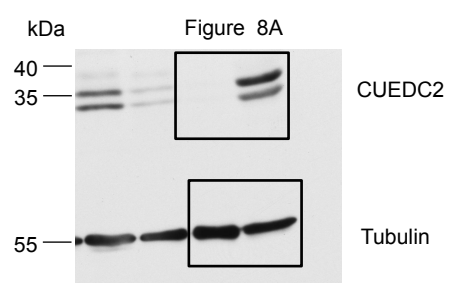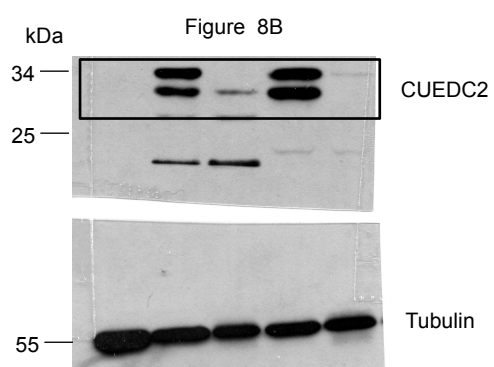

Supplement: Supplementary file 8 — Source Data for Figure 8 [file EMMM-8-813-s007.pdf]
